# Fifteen years of the Protein Crystallography Station: the coming of age of macromolecular neutron crystallography

**DOI:** 10.1107/S205225251601664X

**Published:** 2017-01-01

**Authors:** Julian C.-H. Chen, Clifford J. Unkefer

**Affiliations:** a Bioscience Division, Protein Crystallography Station, Los Alamos National Laboratory, Los Alamos, NM 87545, USA; b Department of Chemistry and Biochemistry, The University of Toledo, Toledo, OH 43606, USA

**Keywords:** neutron crystallography, Protein Crystallography Station, Los Alamos Neutron Scattering Center, H atoms, enzyme mechanisms

## Abstract

The Protein Crystallography Station (PCS), located at the Los Alamos Neutron Scattering Center (LANSCE), was the first macromolecular crystallography beamline to be built at a spallation neutron source. Following testing and commissioning, the PCS user program was funded by the Biology and Environmental Research program of the Department of Energy Office of Science (DOE-OBER) for 13 years (2002–2014). The PCS remained the only dedicated macromolecular neutron crystallography station in North America until the construction and commissioning of the MaNDi and IMAGINE instruments at Oak Ridge National Laboratory, which started in 2012. The instrument produced a number of research and technical outcomes that have contributed to the field, clearly demonstrating the power of neutron crystallo­graphy in helping scientists to understand enzyme reaction mechanisms, hydrogen bonding and visualization of H-atom positions, which are critical to nearly all chemical reactions. During this period, neutron crystallography became a technique that increasingly gained traction, and became more integrated into macromolecular crystallography through software developments led by investigators at the PCS. This review highlights the contributions of the PCS to macromolecular neutron crystallography, and gives an overview of the history of neutron crystallography and the development of macromolecular neutron crystallography from the 1960s to the 1990s and onwards through the 2000s.

## Abstract 2

This article highlights scientific and technical contributions from the Protein Crystallography Station at Los Alamos, the first purpose-built macromolecular crystallography station at a spallation neutron source.

## 1. H atoms in macromolecules and neutron scattering   

Nearly one-half of all atoms in a macromolecule are H atoms, and these H atoms play essential roles in macromolecular structure and catalysis. Hydrogen bonds are one of the most basic of intermolecular and intramolecular interactions. They are critical components of protein secondary structure, forming the basis for α-helices and β-sheets. In nucleic acids, base pairing is mediated by hydrogen bonds. Hydrogen bonds between ligand or solvent molecules and proteins are key parts of the entropic and enthalpic terms that define the strength and stability of molecular interactions. Despite their importance, however, H atoms are very difficult to visualize experimentally in X-ray crystal structures; as a result, their positions are usually assumed and modeled. Furthermore, species such as H^+^ and highly polarized H atoms, and mobile H atoms with relatively high *B* factors, often found in enzyme active sites, are invisible to X-rays.

X-ray scattering is proportional to the number of electrons in the system. A heavy atom, such as iron (26 electrons), will thus scatter electrons far more strongly than will hydrogen (one electron). Locating H atoms in a protein is very difficult, as the scattering contribution from H atoms is overshadowed by that from neighboring heavier atoms. In the case of proteins, H atoms are normally bonded to C, N, O and S atoms, or found in water molecules tightly associated with the protein. Ultrahigh-resolution X-ray structures, diffracting to better than 1.2 Å resolution, have the ability to determine a limited number of H-atom positions, normally appearing as *F*_o_ − *F*_c_ difference peaks. In practice, even in the highest resolution macromolecular structures reported, such as the high-potential iron–sulfur protein from *Thermochromatium tepidum* at 0.48 Å resolution (PDB entry 5d8v; Hirano *et al.*, 2016 ▸), and crambin, reported at 0.48 Å resolution (PDB entry 3nir; Schmidt *et al.*, 2011 ▸), with a recent data set collected to 0.38 Å resolution (Rosenbaum *et al.*, 2015 ▸), it is possible to locate only about two-thirds of the H atoms in a particular structure. Most of these visible H atoms are in well ordered regions of the protein. H atoms in solvent molecules associated with proteins are normally invisible, although they often play critical functional roles. Similarly, H atoms in active sites are often mobile and cannot be visualized in X-ray structures.

Unlike X-rays, which interact with the electron cloud surrounding the atom, neutrons interact with the atomic nuclei themselves. Generally speaking, while neutrons are scattered by the elements in the periodic table to a similar extent, they can exhibit strong isotope discrimination (https://www.ncnr.nist.gov/resources/n-lengths/). Of relevance to macromolecular crystallography, hydrogen has two stable isotopes, ^1^H and ^2^H (D), which scatter neutrons in a very different manner (Table 1 ▸). The neutron scattering length of ^1^H is −3.74 fm, and there is a large incoherent scattering cross-section, which leads to high noise in neutron scattering. Deuterium (D) has a neutron scattering length of +6.67 fm, which is on a par with other heavier elements found in macromolecules, such as C, N, O and P. This property renders D atoms visible even at moderate resolution (2.5 Å and better). By exchanging labile H atoms for D atoms and assaying their relative occupancies, it is also possible to obtain a map of the dynamics of proteins within the crystalline environment. Furthermore, by assaying the relative occupancy of H and D in critical amino acids, it is also possible to obtain information about the local environments in enzyme active sites and the protonation states of amino acids and solvent molecules in the active sites. This allows the mechanism of enzymes to be much better understood. This has arguably been the major contribution of macromolecular neutron crystallography, with the structures of metalloenzymes and several classes of proteases solved using neutron diffraction.

Neutron and X-ray crystallography are complementary methods for structure–function studies of macromolecules, with X-rays useful in the location of heavy atoms and neutrons useful in determining the location of H atoms and in inferring dynamics.

As noted, the elements have roughly similar neutron scattering properties across the periodic table (Table 1 ▸). In theory, this means that H atoms are more straightforward to locate, which allows the possibility of properly orienting solvent molecules within a structure. However, as neutrons interact with atomic nuclei, the very small size of atomic nuclei compared with the size of the electron cloud surrounding the atom makes the probability of a productive interaction very low. Neutron fluxes at current sources are exceedingly low (2 × 10^6^ n cm^−2^ s^−1^ on the sample at the PCS) compared with third-generation synchrotron facilities such as the APS, NSLS-II and MAX IV (10^11^–10^13^ photons s^−1^). Furthermore, as uncharged particles, neutrons are difficult to detect. Only a small number of elements and isotopes have sufficient cross-sections to detect interactions with neutrons, *e.g.* ^3^He and ^10^B, and secondary reactions are required to generate a measurable phenomenon (light, charge). These factors all contribute to exceptional sample requirements for neutron crystallo­graphy, such as large sample sizes and long measurement times, making it a technically demanding endeavor. For protein crystals, purification of gram quantities of the protein is often needed. For neutron crystallography, protein crystals of greater than 0.1 mm^3^ in size are preferred, 4–5 orders of magnitude larger than those used in synchrotron data collection. Measurement times range from days to months at neutron sources, compared with less than 15 min for a typical synchrotron X-ray diffraction data set (Munshi *et al.*, 2012 ▸). The number of instruments worldwide has also been a limitation. For many years, the only suitable instruments were located at Brookhaven, the National Institute of Standards and Technology (NIST) and the Institut Laue–Langevin (ILL) in Grenoble, with no protein crystallography stations after the closure of the High Flux Beam Reactor (HFBR) at Brook­haven until the commissioning of the LADI instrument at ILL in the mid-1990s. Nevertheless, investments in improving neutron crystallography over the past two decades have led to major advances with regards to sample requirements and data-collection times, in addition to more beam time and instruments being available.

## 2. A brief history of macromolecular neutron crystallography   

**Figure 1:**
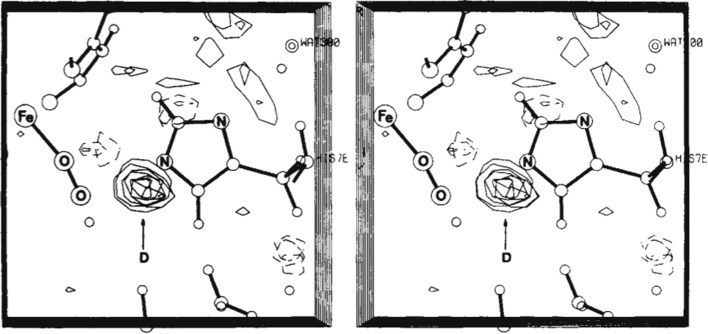
Difference nuclear density demonstrating the protonation of N^η^ of the heme-coordinating histidine in myoglobin. Reprinted with permission from Nature Publishing Group: Phillips & Schoenborn (1981 ▸), *Nature (London)*, **292**, 81–82. Copyright (1981) Nature Publishing Group.

Several comprehensive reviews of macromolecular neutron crystallography have been published in the last few years (Blakeley, 2009 ▸; Schoenborn, 2010 ▸; Niimura & Podjarny, 2011 ▸; Blakeley *et al.*, 2015 ▸); however, a few relevant milestones over the past 50 years are presented here. The 1960s, more than 20 years after the first recorded neutron diffraction from small-molecule crystals, saw the first application of neutron diffraction to protein crystals in a study on the iron-storage protein myoglobin by Benno Schoenborn, who was initially working at the Medical Research Council Laboratory of Molecular Biology (MRC) in the laboratory of John Kendrew (a Nobel Prize winner in 1962) and then later at Brookhaven National Laboratory. The myoglobin data were measured at the HFBR at Brookhaven. It took nearly a year to record 4800 reflections, yielding a 2.8 Å resolution map that clearly showed the protonation of the N^η^ of the imidazole ring on the histidine, coordinating the FeO_2_ in oxymyoglobin (Fig. 1 ▸; Schoenborn, 1969 ▸; Phillips & Schoenborn, 1981 ▸).

**Figure 2:**
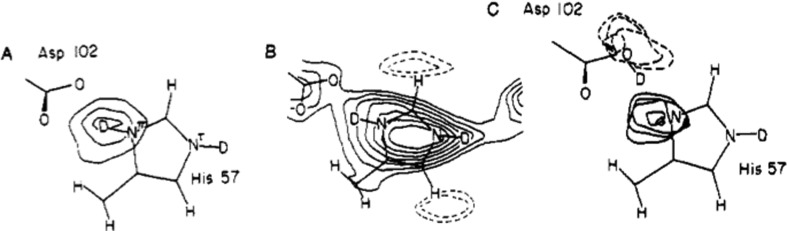
Nuclear density map showing protonation of His57 in trypsin. Left, difference density map showing protonation of N^δ^ of His57. Center, 2*F*_o_ − *F*_c_ nuclear density map with double protonation of His57 and negative density peaks for the unexchanged H atoms. Right, difference density map with the proton modeled on Asp102. The contour shows that the proton belongs on His57 (solid lines) and not Asp102 (dotted lines). Reprinted with permission from Kossiakoff & Spencer (1981 ▸), *Biochemistry*, **20**, 6462–6474. Copyright (1981) American Chemical Society.

The first neutron structures of enzymes, ribonuclease A and trypsin, appeared in 1980 (Wlodawer, 1980 ▸; Kossiakoff & Spencer, 1980 ▸). Trypsin is a prototypical serine protease, a family of universal enzymes that rely on a catalytic triad generally composed of Ser/His/Asp. In human systems, trypsin plays major roles in digestion, but also in cellular processes that, when misregulated, lead to disease states such as cancer. Tony Kossiakoff collected neutron diffraction data at the Brookhaven HFBR, using a single large (∼1 mm^3^) crystal of monoisopropylphosphoryl-trypsin (MIP-trypsin) that had been soaked in deuterated mother liquor for nearly ten years. The neutron structure showed unambiguously that histidine residue 57 (His57) of the catalytic triad (Ser/His/Asp) was doubly protonated, and that the serine residue had a significantly altered p*K*_a_ value, which allowed it to function as a potent nucleophile in the chemical environment of the enzyme active site (Fig. 2 ▸; Kossiakoff & Spencer, 1981 ▸).

A diffractometer was also available at the reactor source at NIST in Gaithersburg, Maryland, USA. Prince, Santoro and Wlodawer modified an existing diffractometer with a position-sensitive ^3^He-based detector (Prince *et al.*, 1978 ▸). Wlodawer and coworkers published neutron structures of ribonuclease A (Wlodawer, 1980 ▸; Wlodawer *et al.*, 1983 ▸; Wlodawer & Sjölin, 1983 ▸), bovine pancreatic trypsin inhibitor (BPTI; Wlodawer *et al.*, 1984 ▸) and insulin (Wlodawer *et al.*, 1989 ▸). Recognizing that neutron data were often incomplete and weak, Wlodawer and Hendrickson developed methods of using X-ray diffraction data in parallel with neutron data to aid in the refinement of the structures (Wlodawer & Hendrickson, 1982 ▸).

In Europe, neutron diffraction studies on the triclinic form of lysozyme, focusing on the binding of solvents such as ethanol and DMSO, were performed on the D8 and D19 instruments at the Institut Laue–Langevin reactor facility (Lehmann & Stansfield, 1989 ▸; Lehmann *et al.*, 1985 ▸). The structure of the vitamin B_12_ coenzyme and its solvent structure were also studied by neutron diffraction using the D8 instrument (Savage, 1986 ▸; Langan *et al.*, 1999 ▸).

Wlodawer, Kossiakoff and others also used neutron crystallography to study the dynamics of proteins in a crystalline environment, taking advantage of the different scattering properties of the H and D isotopes, and monitoring the degree of H/D exchange in the protein amide backbone (Wlodawer & Sjölin, 1982 ▸; Kossiakoff, 1982 ▸, 1984 ▸; Mason *et al.*, 1984 ▸). By utilizing H/D exchange at exchangeable hydrogen positions in side chains, it was possible to determine the conformations of methyl rotors and tyrosine hydroxyl groups, indicating that the hydrogen positions were much more constrained than originally thought (Kossiakoff & Shteyn, 1984 ▸; Kossiakoff *et al.*, 1990 ▸). By using D_2_O–H_2_O difference maps, it became possible to determine and orient the conformations of solvent molecules hydrating the protein structure (Harrison *et al.*, 1988 ▸; Kossiakoff *et al.*, 1992 ▸; Finer-Moore *et al.*, 1992 ▸; Bon *et al.*, 1999 ▸). These were fundamental findings about protein structure and side-chain conformations that have been useful in many disciplines, including computational chemistry, molecular dynamics, drug design and quantum chemistry (McDowell & Kossiakoff, 1995 ▸).

The HFBR at Brookhaven was shut down between 1989 and 1991, and again starting in 1997, before being permanently closed in 1999. Benno Schoenborn was recruited to Los Alamos National Laboratory (LANL) in 1993 to design and inaugurate a new macromolecular neutron crystallography station at the Lujan Center spallation neutron source. In Europe, the reactor at the Institut Laue–Langevin (ILL) in Grenoble was shut down for a five-year period starting in 1990. During this time, the LADI instrument was upgraded to a neutron imaging plate and was used for data collection after the restart of the reactor in 1995 (Niimura *et al.*, 1997 ▸; Langan *et al.*, 1999 ▸). As such, little neutron protein crystallography work was conducted during the early 1990s; however, advances in molecular biology and automation revolutionized macromolecular X-ray crystallography during this decade and led to a resurgence of interest in neutron crystallography.

**Figure 3:**
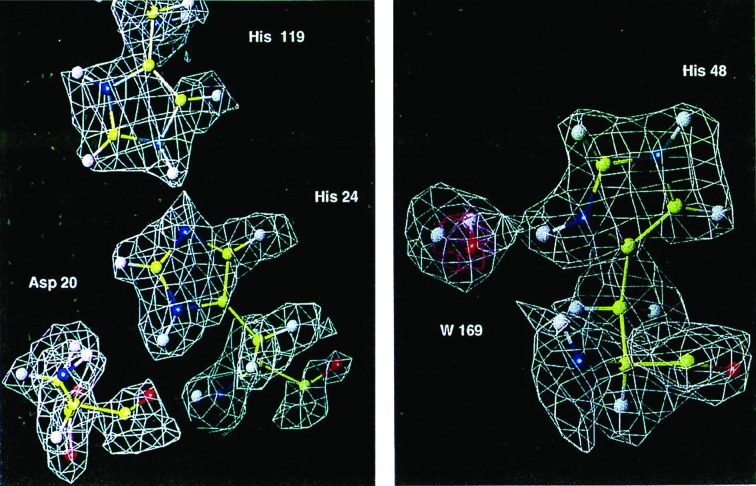
Nuclear density for perdeuterated myoglobin, showing positive density for nonexchangeable H (D) atoms. Reprinted with permission from Shu *et al.* (2000 ▸), *Proc. Natl Acad. Sci. USA*, **97**, 3872–3877. Copyright (2000) National Academy of Sciences.

Until the early 1990s, all reported macromolecular neutron structures utilized protein isolated from natural sources. The advent of commercialized molecular biology and new tools for the overexpression of proteins of interest in *Escherichia coli* facilitated the attainment of the large quantities of purified protein needed for neutron crystallography. The first neutron structure reported using recombinantly expressed protein was a subtilisin BPN′ mutant (Kossiakoff *et al.*, 1991 ▸). Production of fully deuterated (perdeuterated) proteins also relied on molecular biology and knowledge of bacterial biosynthetic pathways. Perdeuterated staphylococcal nuclease and myoglobin were expressed for the purposes of neutron crystallography (Gamble *et al.*, 1994 ▸; Shu *et al.*, 1996 ▸), leading to a neutron structure of perdeuterated myoglobin as a proof of principle (Fig. 3 ▸; Shu *et al.*, 2000 ▸).

In the 1990s, macromolecular X-ray crystallography was becoming increasingly streamlined through hardware (synchrotrons, fast-readout CCD detectors, robotics, cryocooling), software (integrated crystallographic refinement programs) and easily accessible crystallization (high-throughput screening, ready-made crystallization screening kits). These developments opened up a wide range of proteins for structural biology studies, and helped drive a new demand for neutron diffraction instruments and more efficient data collection.

The Protein Crystallography Station (PCS) was designed and built to address these difficult problems, offering a state-of-the-art, high-efficiency detector located at a pulsed spallation neutron source to reduce sample-measurement times through the production of a spectrum of resolvable time-of-flight (TOF) neutrons (Langan & Greene, 2004 ▸; Langan *et al.*, 2004 ▸, 2008 ▸). Initial design of the instrument was completed in November 1996 (Schoenborn, 1996 ▸), and the instrument was commissioned in 2001–2002. During the user program of the PCS, a great deal of effort was invested in utilizing deuteration as a means of reducing the sample-size requirement and the measurement times for neutron samples. A deuteration facility was maintained for users to produce fully and partially deuterated proteins for crystallization using *E. coli*-based expression systems. Although no perdeuterated neutron structures were reported from the PCS, the facility successfully produced several perdeuterated proteins and X-ray structures (Liu *et al.*, 2007 ▸; Blum *et al.*, 2010 ▸). Neutron diffraction data from perdeuterated and selectively deuterated protein samples have been collected at LADI-III at the ILL (Blakeley *et al.*, 2010 ▸) and on the monochromatic instrument D19 (Cuypers *et al.*, 2013 ▸, 2016 ▸; Haupt *et al.*, 2014 ▸), demonstrating that deuteration can clearly shorten data-collection times, reduce the size of suitable crystals and increase the visibility of H atoms. The protocols and methods employed to deuterate proteins are well documented, have led to successful structure determinations of otherwise difficult targets and have become an essential part of neutron sources worldwide (Hazemann *et al.*, 2005 ▸; Petit-Haertlein *et al.*, 2009 ▸, 2010 ▸; Tomanicek *et al.*, 2011 ▸; Howard *et al.*, 2011 ▸, 2016 ▸; Munshi *et al.*, 2012 ▸; Cuypers *et al.*, 2013 ▸, 2016 ▸; Meilleur *et al.*, 2013 ▸; Weber *et al.*, 2013 ▸; Haupt *et al.*, 2014 ▸; Gerlits *et al.*, 2016 ▸; Haertlein *et al.*, 2016 ▸). In addition, computational tools were developed to simplify and integrate neutron crystallography into available programs and software suites for streamlined macromolecular crystallography (much of it developed at other DOE facilities). This multi-disciplinary approach has effectively revolutionized macromolecular neutron crystallography over the past decade, with the PCS strongly contributing to these developments. To date, this includes 21 structures in the Protein Data Bank (PDB) based on data collected at the PCS (Table 2 ▸).

Indeed, the work performed at the PCS during the 13 years of the DOE-funded user program revealed both unexpected and critical data on enzyme mechanisms and highlighted the unique characteristics and chemistry of enzyme active sites. These data have proved to be critical for drug design and protein engineering, and can be utilized with technologies to yield significant human and economic benefits. To date, although structures determined using neutron diffraction still represent a very small fraction (106 structures) of PDB entries (∼120 000 structures), these structures represent the failure of any other probe, be it X-rays, NMR or the use of electrons, to answer a structural biochemistry question, and which neutron diffraction could provide. Macromolecular neutron crystallo­graphy therefore occupies a very special place in structural molecular science.

## 3. Description of the Protein Crystallography Station   

The PCS is located on Flight Path 15 (FP15) emanating from Target 1 at the Lujan Center, part of a suite of instruments making use of neutrons produced upon the bombardment of a heavy-metal target with high-energy protons. This process produces neutrons that are ejected from the metal target, called spallation neutrons. Spallation neutrons have a distinct advantage over neutrons produced by a reactor source in that they can be produced in bursts. The produced spallation neutrons vary in energy, with the largest population around 1–3 MeV. The de Broglie equation
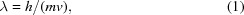
where the mass of a neutron *m* is 1.6749 × 10^−27^ kg, relates the wavelength and velocity of the neutrons. Over a distance of 28 m, the target-to-detector distance for the PCS, neutrons of wavelength 0.6–6 Å arrive over a period of 4.5–45 ms, with the highest energy (shortest wavelength) neutrons arriving at the detector first. With the 20 Hz pulse frequency, a packet of neutrons is generated from the target every 50 ms, which dictates the design of the flight paths. Nearest the target is a beryllium reflector to direct neutrons towards the beam pipe, and also a water moderator, where the thermal neutrons are generated.

**Figure 4:**
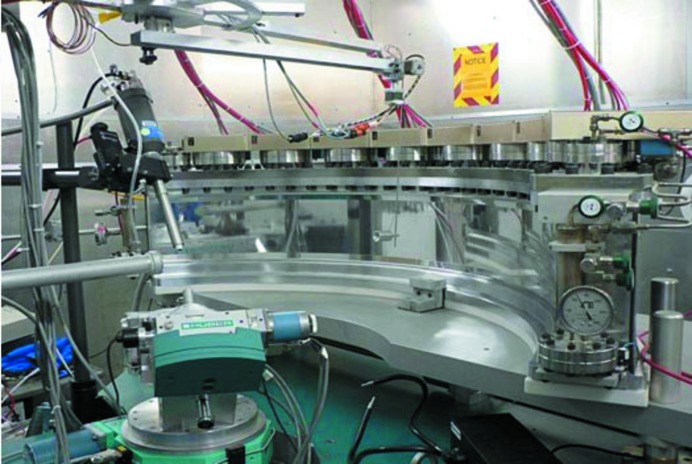
Overview of the PCS detector environment. On the left is the beam pipe, Oxford Cryosystems cryocooling arm and kappa goniometer. On the right is the ^3^He detector system.

For the PCS, a chopper system selects neutrons in the wavelength range 0.6–6 Å and filters out high-energy, shorter-wavelength neutrons and gamma rays that may be potentially damaging to biological systems and that may shorten the lifetime of the samples. The rotation of the chopper is phase-locked with the 20 Hz pulse frequency of the neutron pulses. The distance from the target to the sample is 28 m. A curved detector, with 120° of coverage, lies 70 cm from the sample. To record the highest resolution reflections, the 2θ arm can be moved, allowing nearly 360° of coverage. The detector uses ^3^He to detect neutrons and provides readout in real time (Fig. 4 ▸). A more detailed description follows.

### 3.1. Target/moderator/reflector/shielding   

**Figure 5:**
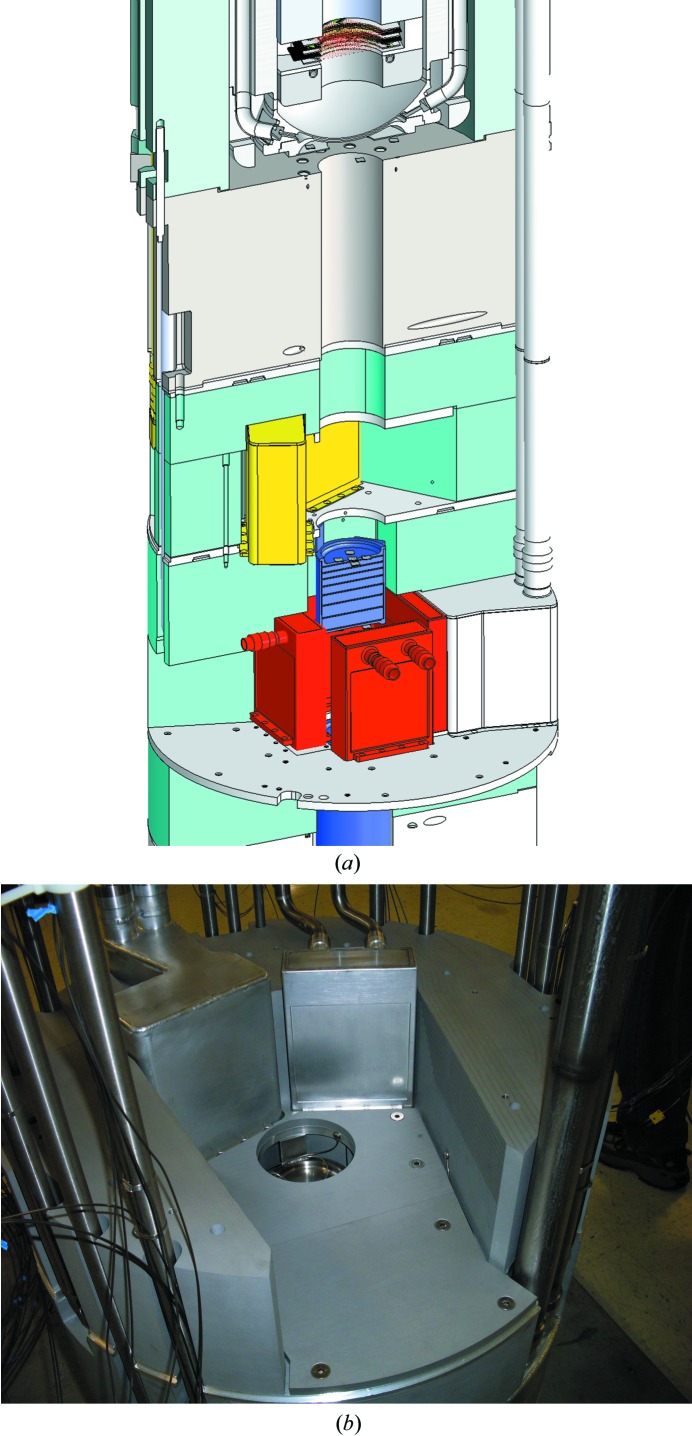
(*a*) Schematic of the Mark-III TMRS. Beryllium reflectors are in cyan, tungsten target plates in blue and moderator suites in yellow and red. The proton beam strikes the target from above. (*b*) Upper moderator suite, with the H_2_O moderator for FP14 and FP15 at the center, a H_2_ moderator to its left and beryllium reflectors arranged around the moderator. The neutrons for FP15 are directed towards the foreground.

A proton beam accelerated to 84% of the speed of light (800 MeV) bombards a tungsten target, leading to neutrons being ejected from the target. The facility operates at around 100 kW power. Currently in place at Lujan Center is a fourth-generation, integrated target/moderator/reflector/shielding assembly (Mark-III TMRS), which was installed in mid-2010 (Fig. 5 ▸). The measured neutron flux became threefold higher after target replacement and installation of the Mark-III TMRS, estimated at 2 × 10^6^ n cm^−2^ s^−1^ (Mocko *et al.*, 2011 ▸; Mocko & Muhrer, 2013 ▸; M. Mocko, personal communication). To accommodate the desired spectral characteristics of the suite of instruments, six different moderators are arranged within the unit on two levels, using both liquid H_2_ and H_2_O as the moderator medium. The target consists of a series of stacked tungsten plates, clad with tantalum to contain the spallation byproducts and cooled with circulating water. For the PCS, a coupled water moderator is used, with the water circulated inside a 13 × 13 × 4 cm container and surrounded by a beryllium block that acts as a neutron reflector. The entire TMRS assembly is compact, measuring 60 cm in diameter and 3 m in height. This is inserted into a crypt that contains a beam stop and a vacuum steel casing. In turn, the crypt is surrounded by the biological shielding, which extends 3 m. The choice of moderator and reflector is critical; wavelength resolution is achieved at the expense of neutron flux and *vice versa*. For the PCS, it was important to have a high flux of neutrons, as the relatively long 28 m flight path and desired crystal diffraction limits could partially offset the ambiguities of wavelength resolution.

### 3.2. Shutter, chopper and collimation   

The shutter consists of a mercury reservoir that fills a section of the beam pipe by gravity. The shutter is opened by pumping He gas to move the mercury back into the reservoir, such that the section of the beam pipe is filled with He during data collection. The target, moderator and shutter are all contained within the 3 m biological shielding surrounding the target. More powerful spallation sources such as the Spallation Neutron Source (SNS; 1 MW) require thicker shielding.

A composite T0/T1 chopper is located 9.5 m downstream of the target. It restricts the wavelength of neutrons to the 0.6–6 Å range. The T0 component further removes any high-energy neutrons and residual gamma rays that may damage biological samples. The T1 component removes the so-called ‘frame overlap’, ensuring that the long-wavelength, slowest-traveling neutrons arrive at the detector (45 ms) before the start of the next neutron pulse (50 ms).

Normally, T0/T1 choppers are designed on separate rotors. In the case of the PCS, a single blade of 30 cm thickness and 30 cm radius acts as the T0 unit, while the T1 is the chopper housing that runs under helium to reduce wear on the bearings and is painted with neutron-absorbing boron paint. The rotation of the chopper is synchronized with a 20 Hz repetition rate of the pulse to stop overlap of radiation from the previous pulse. It is also tuned to the efficiency of the detector (which is highest in the 1–2 Å range).

The collimation was designed with the intention of producing a neutron beam with a size of ∼5 mm and a divergence of approximately 0.12°, matching that of an average protein crystal at room temperature. This has been accomplished by the placement of 16 boron ceramic disks (scrapers) with circular apertures that gradually narrow the neutron beam from the initial 13 × 13 cm size when it exits the moderator. Three disks (boron carbide) are located within the bulk shielding, while the remaining 13 disks (boron nitride) are in the beam pipe. The beam pipe is operated under vacuum.

Additional hardware were proposed, including a focusing mirror and a tail-cutting device for further refinement of the neutron spectrum, but were not constructed (Schoenborn, 1996 ▸; Langan *et al.*, 2001 ▸).

### 3.3. Goniometer, detector and software   

The goniometer is a custom-built Huber model with a quasi-three-circle setting, allowing manipulation of the crystal orientation in the φ, ω and κ orientations, in addition to the *z* (vertical) translation. This allows wide sampling of crystal orientations without moving the detector. The goniometer can be controlled from within the hutch and also remotely by computer; the data-acquisition software allows multiple frames to be collected using a single command.

The PCS detector is a curved, ^3^He-based, position-sensitive detector offering real-time readout, built by the Instrumentation Division at Brookhaven National Laboratory (Radeka *et al.*, 1996 ▸; Mahler *et al.*, 1999 ▸; Fried *et al.*, 2002 ▸). The detector is among the most versatile in the world. The use of spallation neutrons at 20 Hz demanded a position-sensitive detector capable of a fast readout. The use of ^3^He also offers a number of distinct advantages over other neutron concepts, including the more recent scintillation-based designs (Anger cameras) currently in use at facilities such as SNS. The detection efficiency of ^3^He is very high; single neutron interactions are detectable. The timing resolution of the electronics is ∼1 ms. In real terms, on the PCS, with its 28 m flight path, the wavelength resolution approaches ∼0.15 Å. The detector is mounted on a table concentric with the goniometer, allowing rotation of the detector by 2θ, in addition to the *z* (vertical) translation of the detector, which is independent of the *z* translation of the crystal. These settings allow the detector to be manipulated to collect high-resolution data. The active surface area of the detector is 150 × 20 cm, split into eight equal segments, spanning an angle of 120°.

The detector uses ^3^He, which interacts with a neutron in the following reaction:
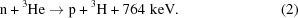
The ^3^H and proton are emitted in opposite directions, with the proton carrying the majority of the kinetic energy (573 keV). The proton range is several centimetres, requiring the addition of a stopping gas to achieve the necessary spatial resolution. In the PCS detector, a small amount of propane is added to the ^3^He chamber, which sharpens the resolution to ∼1 mm. The gas mixture is maintained at ∼7 atm ^3^He and 2.5 atm propane, a proportion that allows ∼50% neutron detection efficiency at 0.6 Å and approaches 100% efficiency at 6 Å. The gas is housed in a special aluminium alloy pressure vessel that yields at 73 000 psi, with an 8 mm thick window that minimizes neutron scattering from the vessel materials. Despite the superior neutron-detection properties of ^3^He, it has very low natural abundance (0.000137%) and is expensive. The gas mixture is internally circulated and is topped off every five years. The scarcity of ^3^He has in part driven the development of the scintillation-based detector technologies currently used on the SNS single-crystal instruments. Table 3 ▸ lists the specifications of the detector.

**Figure 6:**
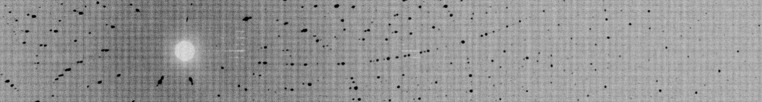
Quasi-Laue projection of time-of-flight neutron diffraction from a crystal of crambin. The detector has been rotated to a 2θ angle of 30° to record high-resolution reflections.

Readout in real time is provided by four DAQs. The system was upgraded in mid-2011, using software written by Marat Mustyakimov that allowed computer control of the gonio­meter. A modified version of d*TREK is used for data integration and processing, and *LAUENORM* is utilized for wavelength normalization of the integrated reflections (Helliwell *et al.*, 1989 ▸; Pflugrath, 1999 ▸; Langan & Greene, 2004 ▸). Software for structure refinement is discussed below (§[Sec sec4.7]4.7). The program can combine the time slices into a quasi-Laue diffraction pattern. An example of such a pattern is shown in Fig. 6 ▸.

## 4. Science highlights   

The user program was funded by DOE-OBER from 2002 to 2014, yielding numerous advances in macromolecular neutron crystallography. In this section, a number of science highlights based on the PCS are described. These include the LANL mission-relevant enzymes d-xylose isomerase (XI), carbonic anhydrase (CA) and diisopropyl fluorophosphatase (DFPase), in addition to enzymes of pharmaceutical interest such as dihydrofolate reductase (DHFR) and endothiapepsin, as well as methods-based work on crambin and joint X-ray and neutron refinement. A total of 21 structures using data collected at the PCS have been deposited in the PDB at the time of writing (Table 2 ▸).

### 4.1. d-Xylose isomerase (XI)   

**Figure 7:**
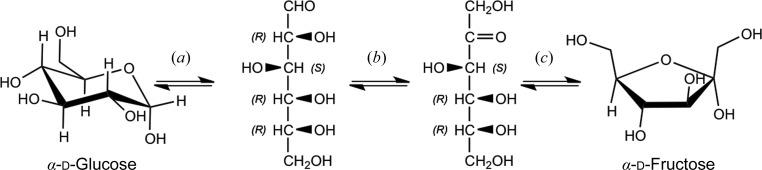
Mechanistic pathway for the isomerization of d-glucose to d-fructose, catalyzed by XI, with ring opening (*a*) followed by isomerization (*b*) and ring closing (*c*) (Kovalevsky *et al.*, 2010 ▸).

The PCS has been used to study the mechanisms of two enzymes of importance to DOE programs in biofuels and renewable energy: d-xylose isomerase (XI) and carbonic anhydrase (CA). XI catalyzes the interconversion between the aldo-sugars d-xylose and d-glucose and the keto-sugars d-xylulose and d-fructose, respectively. XI is a commercially important enzyme that is used in the production of soft-drink sweeteners and also in biofuel production. One problem in the efficient use of biomass derived from cellulose is that a significant portion is in the form of xylose, which cannot be fermented by the yeast *Saccharomyces cerevisiae* in the commercial production of ethanol. On the other hand, d-xylulose can be fermented by *S. cerevisiae*. XI, which is encoded by several fungal and bacterial species, is able to perform this conversion, albeit at a slow rate (Fig. 7 ▸). To further harness its commercial potential, and to guide the engineering of XI to increase its activity and to make it an economically feasible methodology, the mechanism of XI has also been extensively studied through structural and functional characterization. Over the last ten years, multiple neutron structures of XI have been solved. The mechanism relies on two divalent metal ions for activity, with a maximal activity around pH 8.0, which is higher than the acidic (pH <6) conditions of biomass conversion.

**Figure 8:**
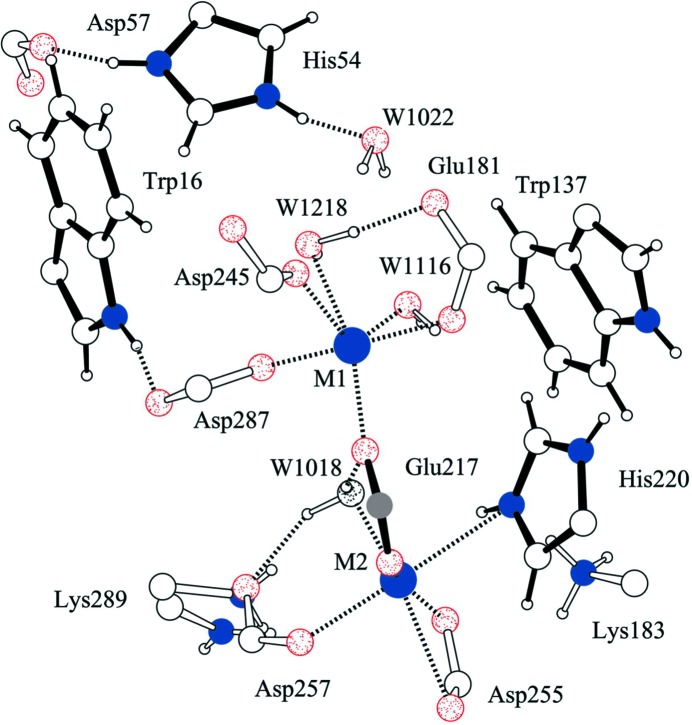
Active-site environment of XI as determined by neutron diffraction, showing the bimetal catalytic center and orientations of water molecules in the vicinity. Reprinted with permission from Katz *et al.* (2006 ▸), *Proc. Natl Acad. Sci. USA*, **103**, 8342–8347. Copyright (2006) National Academy of Sciences.

In a series of studies since 2006, the PCS was used to collect four different states of the enzyme to study the reaction mechanism of XI, including the apoenzyme (Fig. 8 ▸; Katz *et al.*, 2006 ▸), a complex of XI with the product d-xylulose (Kovalevsky *et al.*, 2008 ▸), XI with nickel and a linear sugar resembling an intermediate (Kovalevsky *et al.*, 2010 ▸) and metal-free XI at two pH values (Kovalevsky *et al.*, 2011 ▸). An additional neutron data set was collected at ILL D19 in Grenoble by PCS staff members. Mechanistically, several amino acids, including Asp257, Lys289 and His54, were equivocal in their protonation states even in ultrahigh-resolution X-ray structures. The neutron structures demonstrated that Lys289 was uncharged (deprotonated) and disordered before ring opening and positively charged and ordered after ring opening. His54 was found to be doubly protonated throughout the course of the reaction. The solvent molecule bound to the catalytic metal is found to be water before isomerization and is identified as hydroxide after isomerization. Water orientations and hydrogen bonds are radically shifted during the course of the reaction.

**Figure 9:**
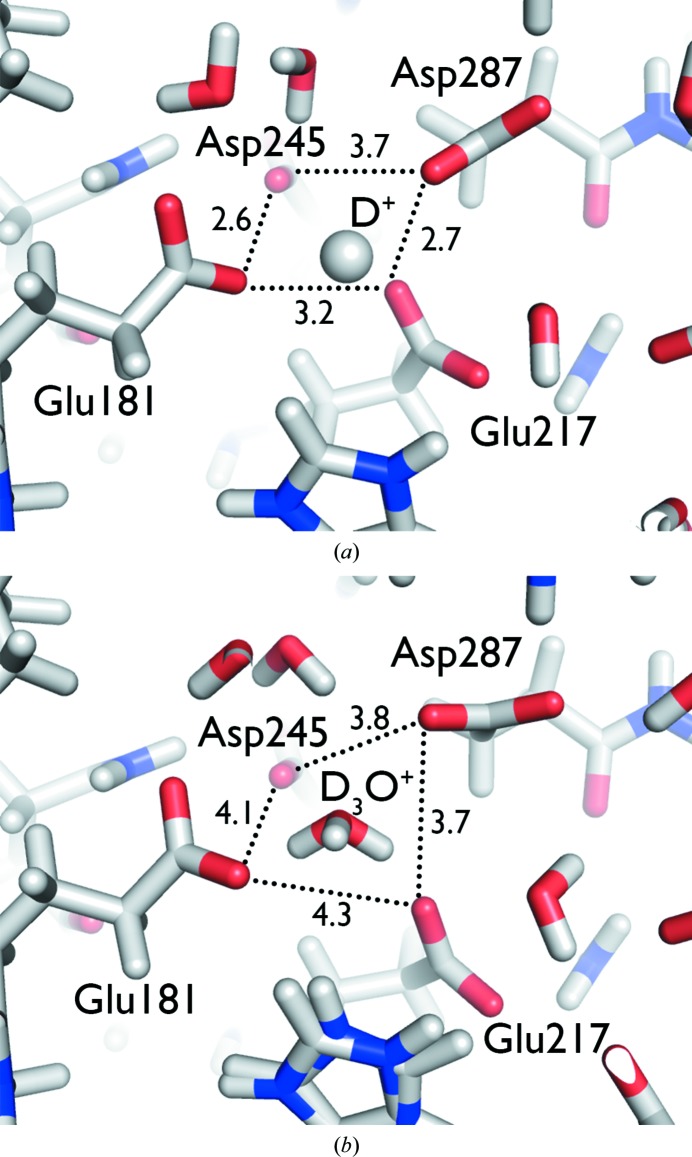
(*a*) Interaction of metal-coordinating residues in XI with D^+^ at pH 5.9 (PDB entry 3qza). (*b*) Interaction of metal-coordinating residues in XI with D_3_O^+^ at pH 7.7 (PDB entry 3kcj) (Kovalevsky *et al.*, 2011 ▸).

One of the major achievements of this work was the ability to use neutrons to resolve and distinguish between the elusive H^+^ and H_3_O^+^ species, the former of which contain no electrons and is therefore invisible in X-ray structures and the latter of which is identifiable as a pyramidal D_3_O^+^ species in nuclear density maps (but appears as a single O atom in electron-density maps). The structure of metal-free XI at pH 7.7 shows a D_3_O^+^ species occupying the site of one of the metals. Under acidic conditions, at pH 5.9, the D_3_O^+^ is dehydrated to a D^+^, with the amino acids collapsing onto the proton (Fig. 9 ▸; Kovalevsky *et al.*, 2011 ▸).

On a technical level, the unit cell of XI is one of the largest ever resolved using neutron diffraction, crystallizing in space group *I*222 with unit-cell parameters *a* = 94 , *b* = 100 , *c* = 103 Å. More recent experiments on another sugar-converting enzyme, levoglucosan kinase, demonstrated resolved diffraction spots from a crystal with a maximum cell edge of 230 Å, exceeding the predicted resolving power of the PCS detector.

### 4.2. Carbonic anhydrase (CA)   

CA is crucial for cellular function and also for green chemistry, as a means of carbon sequestration. CA is a ubiquitous enzyme found in all living organisms, supporting a wide variety of physiological processes. It is a zinc metallo­enzyme catalyzing the interconversion of carbon dioxide to bicarbonate, with a subsequent proton-transfer step. The proton-transfer step is rate-limiting, the excess proton being transferred *via* a water wire that stretches between the catalytic Zn and bulk solvent *via* His64. Like XI, human CA-II has been extensively studied using neutron crystallography (Fisher *et al.*, 2010 ▸, 2011 ▸, 2012 ▸; Michalczyk *et al.*, 2015 ▸). The enzyme is one of the few that work at an apparent *k*_cat_ that is faster than diffusion.

**Figure 10:**
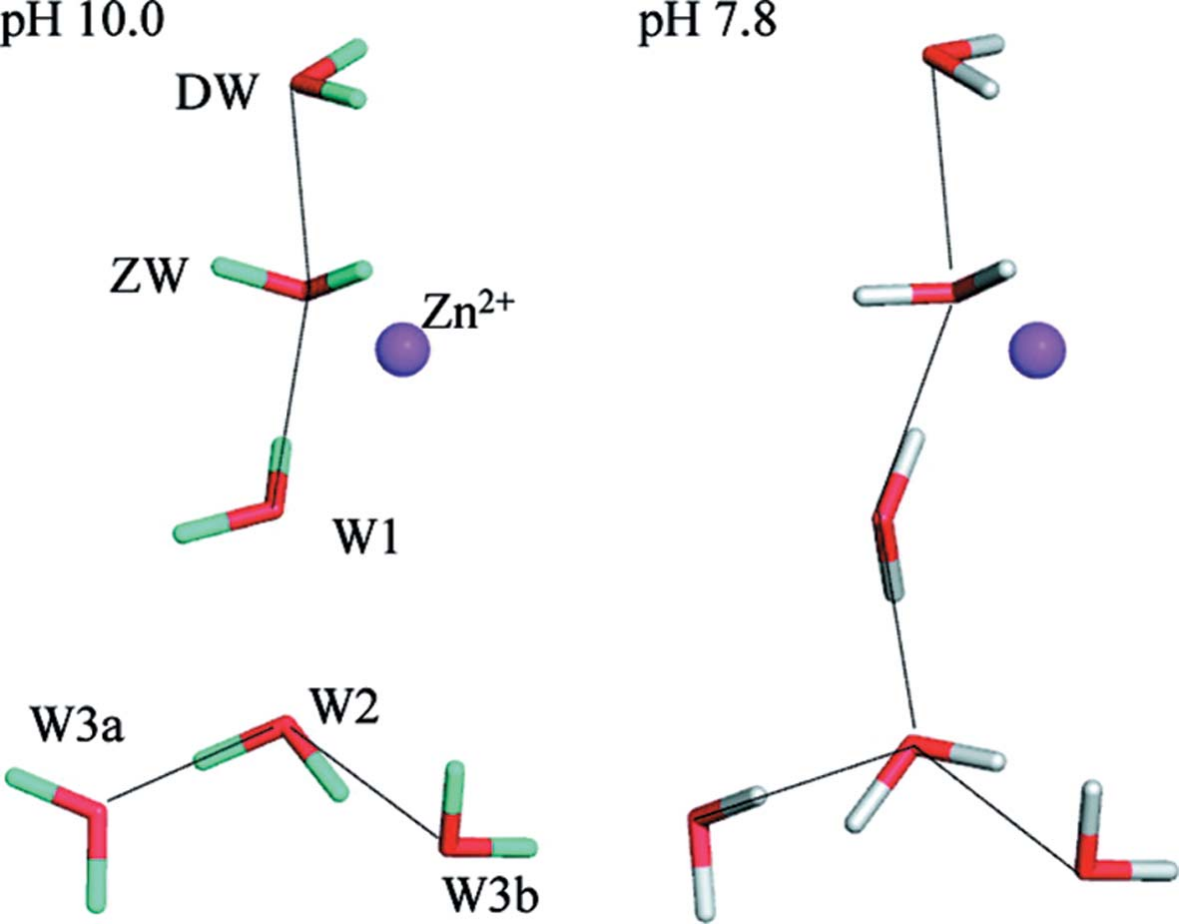
Water-relay network within CA, showing a difference in the orientation of water W1 between pH 7.8 (active) and pH 10.0 (inactive). Reprinted with permission from Fisher *et al.* (2011 ▸), *Biochemistry*, **50**, 9421–9423. Copyright (2011) American Chemical Society.

CA is one of the most extensively studied enzymes using neutron crystallography and a number of critical findings have been discovered that relate to the water relay that moves the excess proton. A single hydrogen bond among a long hydrogen-bonding network was found to act as a switch, explaining the activity of the enzyme at near-neutral and high pH values. Although the positions of the O atoms remained constant, the S atoms were altered in position, and these results could not have been obtained even at the highest X-ray resolutions but were clearly defined using neutron crystallo­graphy. Several findings from the neutron structures of CA have important mechanistic implications. A solvent molecule coordinated to the catalytic metal was identified as water and not hydroxide (Fig. 10 ▸). Enzyme-active sites are often characterized by residues displaying radically altered p*K*_a_ values and unexpected protonation states. The active-site residue His64 was found to be singly protonated and ready to accept a single proton. The hydroxyl group of Tyr7, a residue that helps orient the water network, was found to be deprotonated at pH 10, which was lower than expected. In a recent study jointly using NMR spectroscopy and neutron crystallography, the p*K*_a_ of Tyr7 was determined to be 7.1, which is very low and closely matches that of His64. This low p*K*_a_ is most likely necessary to allow efficient proton transfer in both directions between the Zn ion and the bulk water (Michalczyk *et al.*, 2015 ▸).

**Figure 11:**
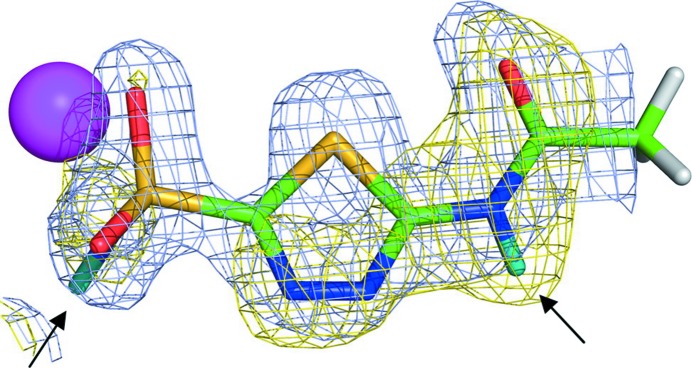
X-ray (gray) and nuclear (yellow) density map for the drug acetazolamide bound to human CA-II. Reprinted with permission from Fisher *et al.* (2012 ▸), *J. Am. Chem. Soc.* **134**, 14726–14729. Copyright (2012) American Chemical Society.

The first neutron structure of a clinical drug–human enzyme complex was solved using data collected at the PCS, showing the anionic form of the diuretic acetazolamide bound to human CA-II (Fig. 11 ▸; Fisher *et al.*, 2012 ▸).

### 4.3. Diisopropyl fluorophosphatase (DFPase)   

Another LANL mission-critical enzyme is diisopropyl fluorophosphatase (DFPase; 315 amino acids, 35 kDa). DFPase is a Ca^2+^-dependent enzyme isolated from the Mediterranean squid head ganglion that is capable of detoxifying a wide range of organophosphorus nerve agents, such as sarin, soman and tabun. These nerve agents act as irreversible inhibitors of acetylcholinesterase (AChE), blocking the conduction of electrical signals at synapses. DFPase functions by hydrolysis of the P—F bond. DFPase is a very stable, heat-tolerant enzyme that can be expressed in large quantities, making it an excellent potential candidate for enzymatic decontamination.

**Figure 12:**
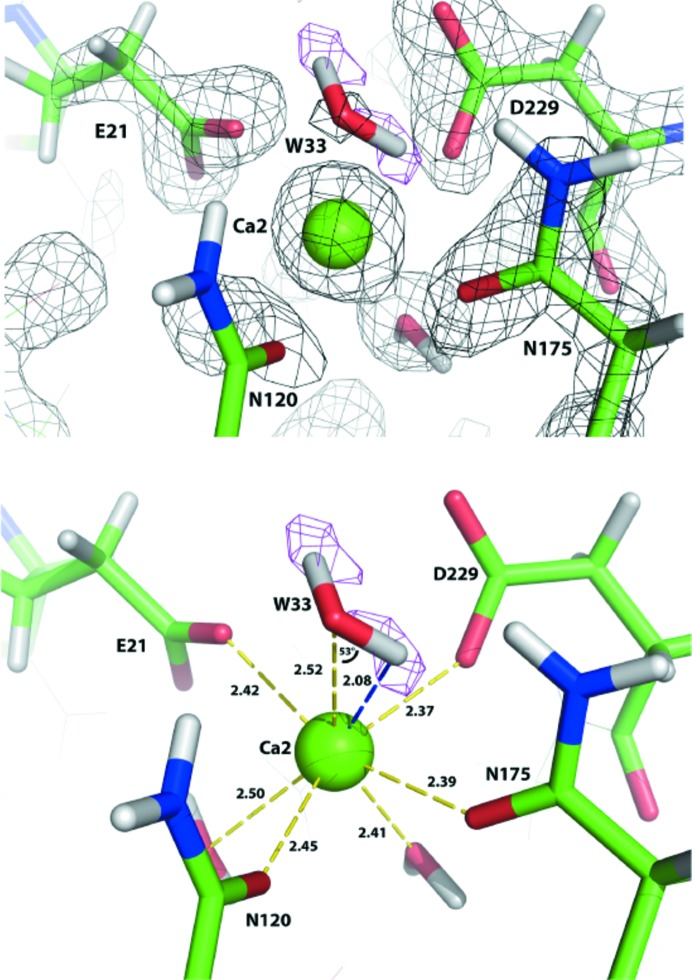
2*F*_o_ − *F*_c_ electron (gray) and nuclear *F*_o_ − *F*_c_ OMIT (magenta) density maps of the DFPase active-site environment. W33 is clearly identified as a water molecule and not a hydroxide, and is situated in an orientation that maximizes hydrogen-bonding interactions. The Ca—O—D angle is 53°. Reprinted with permission from Blum *et al.* (2009 ▸), *Proc. Natl Acad. Sci. USA*, **106**, 713–718. Copyright (2009) National Academy of Sciences.

The remarkable properties of the enzyme have been studied using a variety of chemical and structural techniques. A number of X-ray structures of the wild-type enzyme have been solved and nearly 20 site-directed mutants, including a structure of a substrate analogue (DcPPA)–DFPase complex (Scharff *et al.*, 2001 ▸; Katsemi *et al.*, 2005 ▸; Blum *et al.*, 2006 ▸; Melzer *et al.*, 2009 ▸; Blum & Chen, 2010 ▸). Labeling experiments under single and multiple turnover conditions in ^18^O-labeled water, combined with mass spectroscopy, suggested a mechanism by which a catalytic aspartic acid (Asp229) was involved in direct nucleophilic attack on the P atom of the substrate, creating a phosphoenzyme intermediate (Blum *et al.*, 2006 ▸). However, based on an ultrahigh-resolution X-ray structure (Koepke *et al.*, 2003 ▸), it was also suggested that the mechanism proceeded through a metal-activated water abstracting a proton to create a hydroxide ion that then attacks the P atom. To distinguish these possibilities, the neutron structure of DFPase was solved using a 2.2 Å resolution data set collected at the PCS. The resulting structure showed clearly that an active-site solvent atom coordinated by the catalytic calcium is a water molecule and not a hydroxide (Fig. 12 ▸). The putative nucleophile Asp229 is deprotonated, consistent with the labeling studies (Blum *et al.*, 2009 ▸). Insights from the neutron structure led to the generation of DFPase variants that showed enhanced detoxification properties through engineering stereochemical selectivity to preferably hydrolyze the more toxic stereoisomer of the racemic nerve agents (Melzer *et al.*, 2009 ▸). More recent experiments by the PCS staff, together with the Organization for the Prohibition of Chemical Weapons (OPCW) in The Hague, have demonstrated that the proposed mechanism is conserved within the range of G-type nerve agents.

This was also a technical achievement for the PCS, utilizing one of the smallest crystals at the time for a macromolecular diffraction experiment (0.43 mm^3^) and demonstrating that protein preparation, crystallization and data acquisition could be completed in less than six months (Blum *et al.*, 2007 ▸). A second major achievement in the work on the DFPase structure was the use of joint X-ray/neutron refinement, based on a room-temperature X-ray data set collected from a DFPase crystal together with the neutron data.

### 4.4. Aspartyl proteases   

Aspartyl proteases are among the major protease families, relying on a pair of aspartic acid residues to catalyze cleavage of the peptide bond. Their mechanism has been studied for many decades, in part owing to their importance in numerous physiological and disease processes, such as hypertension and AIDS. Pepsin is a major digestive enzyme, and perhaps the most studied aspartyl protease, HIV-1 protease, recognizes specific cleavage sites on the HIV polyprotein to cleave it into three component enzymes: protease, reverse transcriptase and integrase.

**Figure 13:**
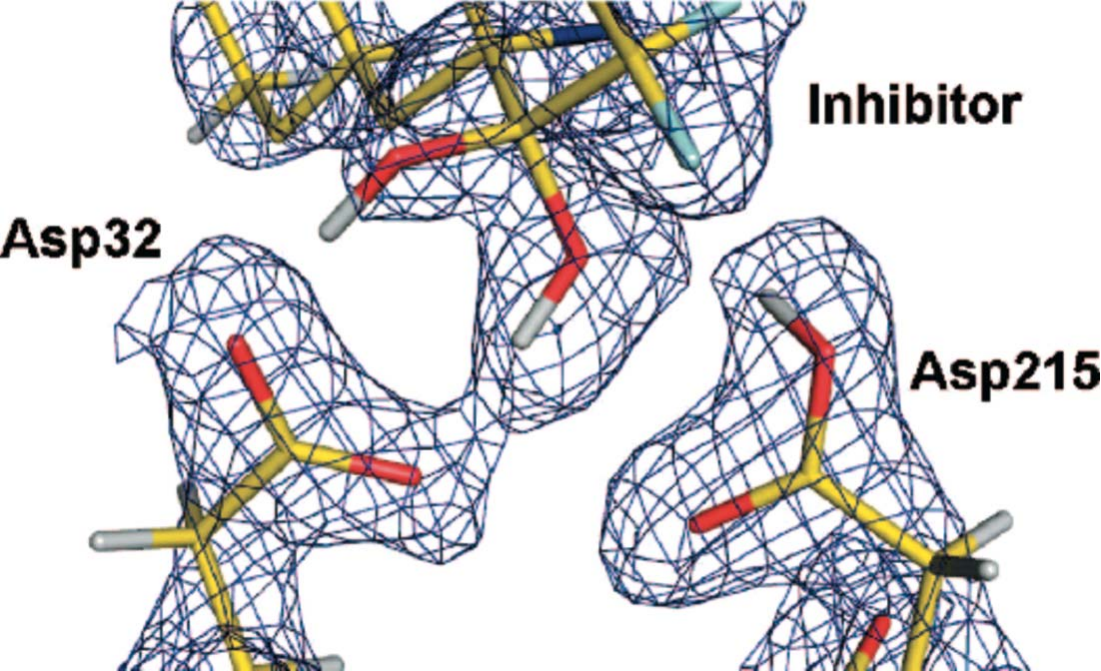
Nuclear density for the active site of the endothiapepsin–inhibitor complex. Asp32 is deprotonated, likely forming a low-barrier hydrogen bond with the *gem*-diol inhibitor, while Asp215 is protonated, acting as a hydrogen-bond donor to the inhibitor. Reprinted with permission from Coates *et al.* (2008 ▸), *J. Am. Chem. Soc.* **130**, 7235–7237. Copyright (2008) American Chemical Society.

Although the aspartyl proteases utilize two aspartic acid residues acting in tandem to cleave the substrate, and many aspartyl proteases such as HIV-1 protease are in fact dimeric, the chemical properties of the aspartic acid residues are not identical. Neutron diffraction data on a crystal of the fungal aspartic protease endothiapepsin in complex with a *gem*-diol inhibitor were collected at the PCS (Fig. 13 ▸; Coates *et al.*, 2008 ▸). Parallel X-ray data sets were also collected at atomic resolution (1.0 Å). Bond lengths were used to infer the protonation state of the aspartate residues after removing stereochemical restraints on the aspartates. The nuclear density maps at 2.0 Å resolution point to one (Asp215) of the two catalytic aspartic acids being protonated, with one of the *gem*-diol H atoms in an orientation and position consistent with a low-barrier hydrogen bond. The stability of the low-barrier hydrogen bond is thought to offset the strain experienced by the scissile peptide bond when productively bound.

### 4.5. Dihydrofolate reductase (DHFR)   

**Figure 14:**
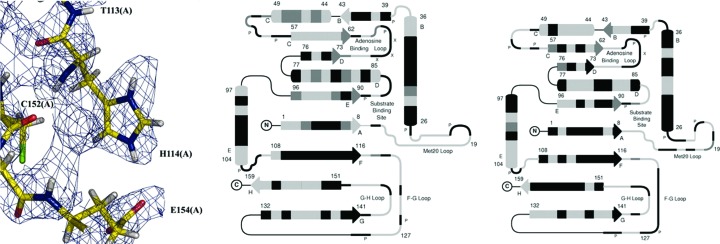
Left, nuclear 2*F*_o_ − *F*_c_ density for active-site residues, showing double protonation of His114. Middle and right, amide backbone H/D-exchange pattern for the two monomers of DHFR, ranging from gray (exchanged) to black (not exchanged). Reprinted with permission from Bennett *et al.* (2006 ▸), *Proc. Natl Acad. Sci. USA*, **103**, 18493–18498. Copyright (2006) National Academy of Sciences.

Another enzyme of medicinal interest, dihydrofolate reductase (DHFR), was studied at the PCS. DHFR is an enzyme that is crucial for numerous biosynthetic pathways, catalysing the NADP-dependent reduction of dihydrofolate (CHF) to tetrahydrofolate (THF). THF in turn is an essential cofactor in the biosynthesis of nucleotides as well as selected amino acids. As such, DHFR is the target of several clinical drugs, such as the chemotherapeutic methotrexate and the antimicrobial agent trimethoprim. These drugs block the ability of the cell to synthesize DNA, ultimately leading to cell death. The neutron structure of the *E. coli* DHFR–MTX complex was solved using data collected at the PCS from a very small crystal of ∼0.25 mm^3^ in volume, revealing binding-induced protonation on the MTX ring at N1 (Fig. 14 ▸; Bennett *et al.*, 2006 ▸). This protonation allows an ionic interaction with an uncharged Asp27. The H/D-exchange pattern in the backbone amides reveals that the two monomers of the dimer have similar, but not identical, H/D-exchange properties.

### 4.6. Crambin   

The small protein crambin (46 amino acids, 4.7 kDa), isolated from the seeds of the Abyssinian cabbage (*Crambe abyssinica*), forms the best ordered macromolecular crystals known, diffracting X-rays to a resolution of 0.38 Å. Although no biological function has been attributed to crambin, the extraordinary diffraction properties of crambin crystals have enabled the development of numerous crystallographic techniques, such as native single-wavelength anomalous diffraction (SAD) phasing (Hendrickson & Teeter, 1981 ▸), direct methods, refinement and also biophysical methods such as molecular dynamics. An early study in the 1980s hinted at the excellent neutron diffraction properties of crambin crystals (Teeter & Kossiakoff, 1984 ▸).

**Figure 15:**
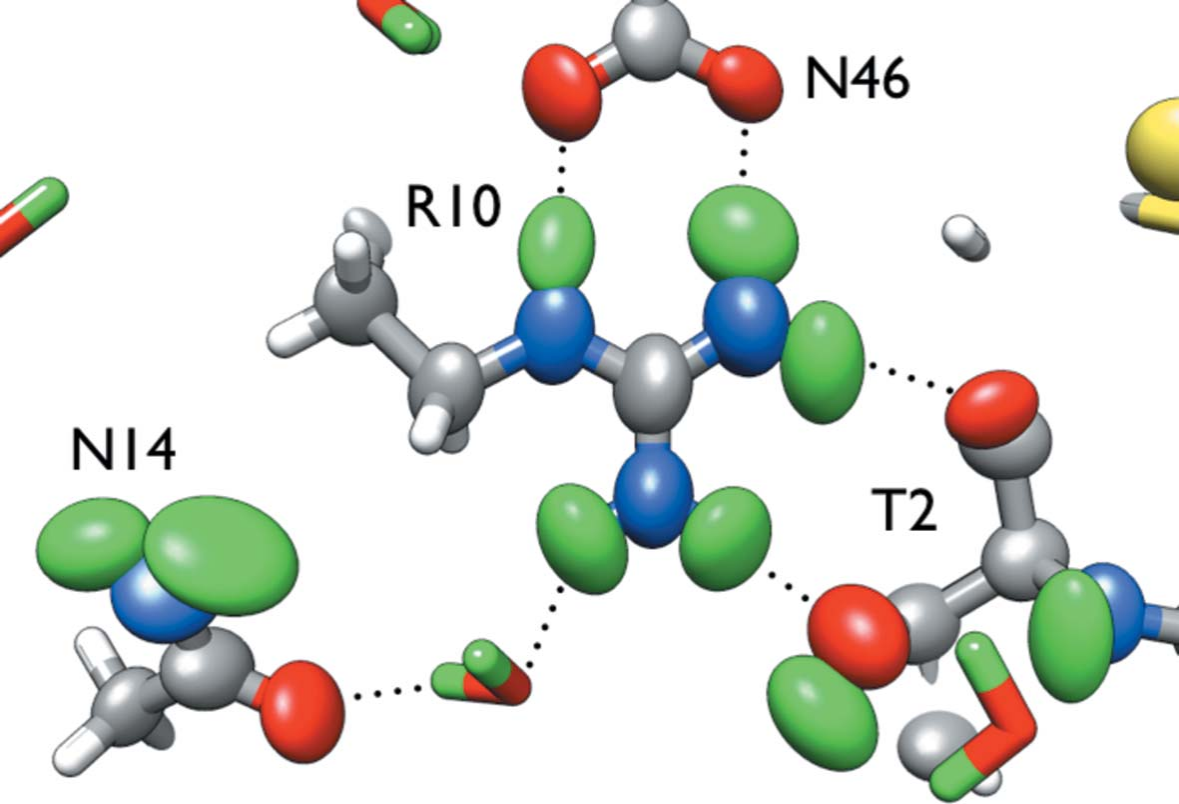
Anisotropic vibrational motions of selected D atoms in crambin. In green are the anisotropic ellipsoids of selected D atoms in crambin, with their respective hydrogen-bonding partners. N atoms are in blue, O atoms in red and C atoms in gray. Reprinted with permission from Chen *et al.* (2012 ▸), *Proc. Natl Acad. Sci. USA*, **109**, 15301–15306. Copyright (2012) National Academy of Sciences.

**Figure 16:**
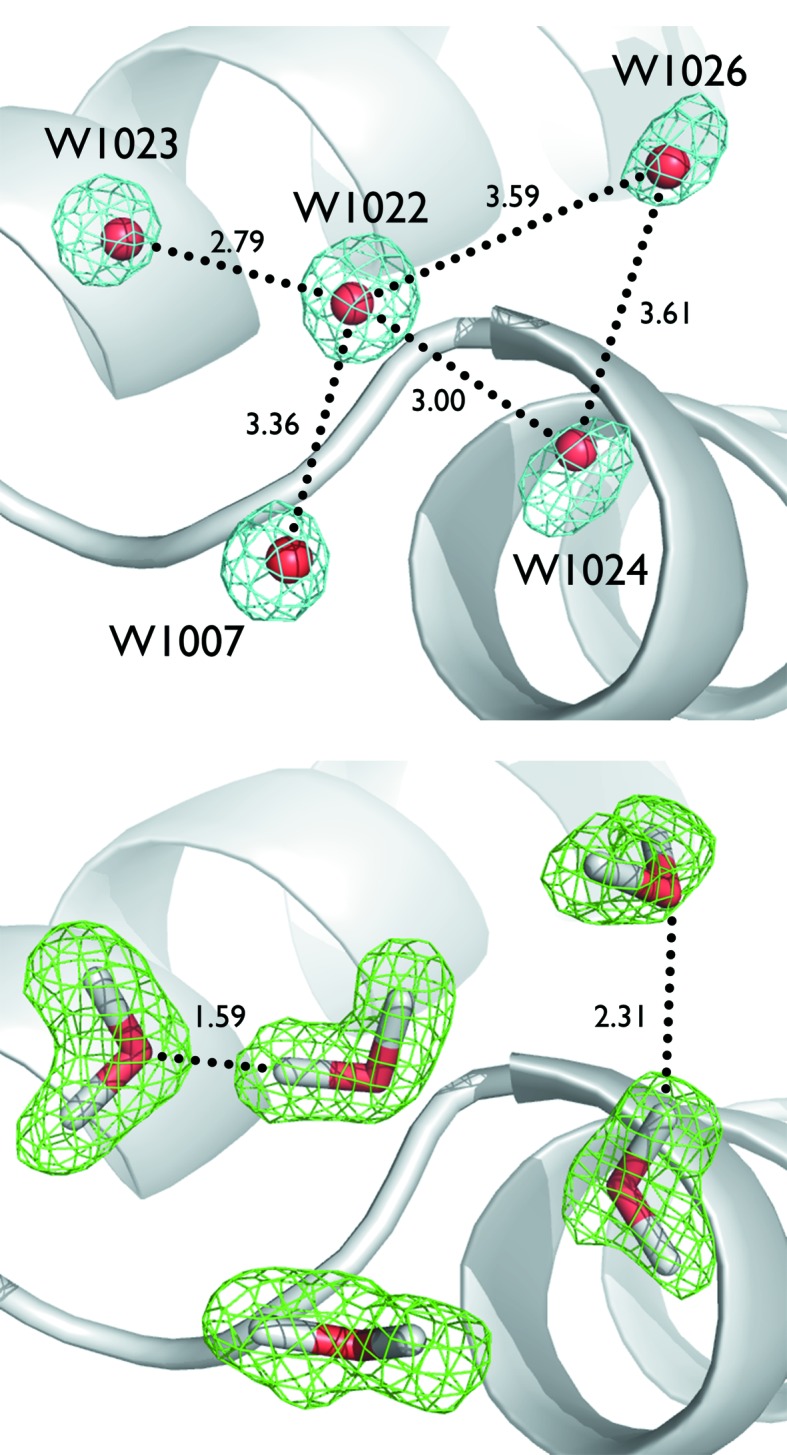
Top, X-ray electron-density map at 0.83 Å resolution showing ambiguous hydrogen-bonding networks in water molecules associated with crambin. Bottom, nuclear density map at 1.1 Å showing the hydrogen-bonding network and clear density for water molecules. Reprinted with permission from Chen *et al.* (2012 ▸), *Proc. Natl Acad. Sci. USA*, **109**, 15301–15306. Copyright (2012) National Academy of Sciences.

A new, complete data set was collected from a large (4 mm^3^) crambin crystal in 2011 at the PCS, yielding observed diffraction beyond 1.0 Å resolution. The unprecedented neutron resolution (85% complete to 1.1 Å resolution) allowed the anisotropic analysis of hydrogen atomic motions in a protein for the first time (Fig. 15 ▸; Chen *et al.*, 2012 ▸). Overall, H atoms are more anisotropic than their bonded neighbors (N, O and S). The work also reinforced the utility of neutron diffraction to elucidate H-atom positions and hydrogen-bonding networks, which are often ambiguous even in the highest resolution X-ray structures (Fig. 16 ▸).

The structure also identified potential C—H⋯O hydrogen-bonding interactions through evidence of partial backbone α-hydrogen H/D exchange. Partial exchange of one of the two backbone H atoms of Gly31 was seen, indicating that the H atoms were in different chemical environments, in agreement with NMR studies. In collaboration with staff from APS, X-ray diffraction data were collected from crambin crystals to an unprecedented resolution of 0.38 Å, the highest resolution recorded for a macromolecule to date. The data collection required a reconfigured beamline and the use of helium-based cryocooling (Rosenbaum *et al.*, 2015 ▸).

Furthermore, the crambin neutron structure yielded perhaps the most accurate structure of the protein and associated solvent atoms to date, which has served as a starting point for theoretical and computational studies on the protein. A number of fundamental questions pertaining to solvent–structure and protein–solvent interactions can be answered by future neutron structures, making crambin an exceptional model system for studying these issues.

### 4.7. Joint X-ray and neutron refinement   

It was recognized early on that the refinement of neutron structures could be problematic. Neutron diffraction data are generally weak and at a lower resolution than X-ray data. Because of the strong scattering from H atoms in addition to the heavy elements (C, N, O, S) in proteins, the number of atomic positions being refined greatly increases, and the data-to-parameter ratio is typically low. Furthermore, as H/D occupancies are refined in neutron structures, overfitting of data is a major concern. The concept of using X-ray diffraction data to complement and supplement neutron diffraction data was developed in detail in the early 1980s in a seminal set of papers by Coppens, implemented for small molecules in *MAUDY* (Coppens *et al.*, 1981 ▸), and by Wlodawer and Hendrickson, implemented in the least-squares crystallo­graphic refinement program *PROLSQ* (Wlodawer & Hendrickson, 1982 ▸). Joint refinement was applied to parallel neutron and X-ray data sets collected from crystals of ribonuclease A (Wlodawer & Sjölin, 1983 ▸), BPTI (Wlodawer *et al.*, 1984 ▸) and insulin (Wlodawer *et al.*, 1989 ▸). In the mid-2000s, joint refinement was incorporated into the commonly used crystallographic refinement programs *CNS* (as *nCNS*) and *PHENIX*. This project was conducted jointly with Lawrence Berkeley Laboratory (LBL) and has continued at ORNL as part of an NIH-funded project (Adams *et al.*, 2009 ▸). The earliest successes of this implementation were the structure refinements of photoactive yellow protein (PYP), endothiapepsin and DFPase using data collected at the PCS (Fisher *et al.*, 2007 ▸; Coates *et al.*, 2008 ▸; Blum *et al.*, 2009 ▸).

This modern implementation of joint neutron and X-ray refinement takes advantage of the maximum-likelihood refinement target function of current crystallographic refinement programs, as opposed to the least-squares target in older programs. In a typical refinement, the two data sets are weighted relative to one another and the progress of the refinement is monitored by *R*_free_ values, and the difference between the *R*_free_ and *R*_work_ values is used to assess the degree of model bias or overfitting.

The joint refinement was found to be especially effective at moderate resolutions in side-chain conformations. In non-perdeuterated structures, the side-chain methyl H atoms (CH_2_/CH_3_) remain unexchanged, and the opposite signs of the scattering lengths of C (+6.64 fm) and H (−3.74 fm) often lead to a ‘cancellation’ effect, with little visible nuclear density, making it difficult to correctly position the side chains. The electron density, on the other hand, is often quite clear. Thus, by using both X-ray and neutron diffraction data in the refinement, it should be possible to more accurately model the side-chain conformation. In terminal groups such as ND_2_ on glutamine and asparagine residues, ND_2_/ND_3_^+^ on lysine residues and the guanidinium group on arginine, D atoms are generally invisible in electron-density maps, but they appear as very strong peaks in nuclear density maps. Similarly, solvent molecules exchanged as D_2_O appear as boomerang-shaped nuclear density, while only the O atom is typically visible in electron-density maps.

The use of electrostatics in joint X-ray/neutron refinement was also investigated using DNA and XI data sets collected at the PCS (Fenn *et al.*, 2011 ▸).

## 5. Conclusions and outlook   

The PCS was designed to take advantage of the time-of-flight neutrons at the Lujan Center spallation neutron source, coupled with a high-sensitivity ^3^He-based detector, to shorten the data-collection period by an order of magnitude. Whereas the first neutron structure of myoglobin involved data collection over the course of a year, it is now possible to collect a complete neutron data set in less than a month. In exceptional cases, using crystals that are perdeuterated, it is now possible to collect entire data sets in 1–3 d (Meilleur *et al.*, 2013 ▸; Munshi *et al.*, 2012 ▸). Refinement of neutron diffraction data has been made easier through joint X-ray/neutron refinement methods in programs such as *CNS* and *PHENIX*.

These practical advances have made neutron crystallo­graphy a more useful tool for understanding protein structure, dynamics and reaction mechanisms. These advances demonstrated at the PCS have helped to drive the development of instrumentation at new beamlines at spallation neutron sources, such as those now available at the SNS, J-PARC and the future ESS. Together with the image-plate technology and the Laue approach driven by work at the ILL (Niimura *et al.*, 1997 ▸; Cipriani *et al.*, 1994 ▸, 1996 ▸; Habash *et al.*, 1997 ▸) and now in use at reactor sources such as HFIR, ILL and FRM-II, data-collection times are now much shorter. In addition, per­deuteration of samples along with these developments means that it is now possible to accommodate larger proteins and study more complex systems (Table 4 ▸; see also Fig. 20 of Blakeley, 2009 ▸).

The science highlights presented in this review demonstrate the utility of neutron crystallography in obtaining useful information about the positions of H (D) atoms at moderate resolution (2.5 Å or better). Deuteration of the exchangeable H atoms in proteins facilitates the study of mobile H atoms in enzyme mechanisms and the interaction of solvent water molecules and their role in catalysis. In exceptional cases, such as crambin, the ultrahigh-resolution neutron protein structure demonstrated that nearly all of the H atoms in a macromolecule can be experimentally ascertained, and the anisotropic vibrational characteristics of D atoms in macromolecules could be observed for the first time.

Over the course of the user program, during which neutrons were available for 4–5 months of the year, the PCS received 220 proposals, with nearly 3000 d of neutron beam time requested, compared with 1418 d of delivered beam. As of the time of writing, 21 structures determined at the PCS have been deposited in the PDB, with additional structures currently in the refinement stage. It is expected that even with the recent commissioning and design of new instruments, neutron beam time is expected to remain at a premium.

## Figures and Tables

**Table 1:** Neutron scattering properties of selected elements in biological systems 1 fm = 10^−15^ m. 1 barn = 10^−24^ cm^2^.

Element	Neutron scattering length (fm)	Neutron coherent scattering cross-section (barns)	Neutron incoherent scattering cross-section (barns)
^1^H	−3.74	1.76	80.27
^2^H (D)	+6.67	5.59	2.05
C	+6.65	5.55	0.001
N	+9.36	11.0	0.5
O	+5.80	4.23	0.0008
P	+5.13	3.31	0.005
S	+2.85	1.02	0.007
Na	+3.63	1.66	1.62
Mg	+5.37	3.63	0.08
K	+3.67	1.69	0.27
Ca	+4.70	2.78	0.05
Mn	−3.73	1.75	0.4
Fe	+9.45	11.22	0.4
Co	+2.49	0.779	4.8
Ni	+10.3	13.3	5.2
Cu	+7.72	7.48	0.55
Zn	+5.68	4.05	0.077
Mo	+6.71	5.67	0.04
W	+4.86	2.97	1.63

**Table 2:** Structures in the PDB collected at the PCS

Protein	Unit-cell parameters (Å, °)	Space group	Resolution (Å)	PDB code	Crystal volume (mm^3^)	Primary citation
Amicyanin	*a* = 27.5, *b* = 56.6, *c* = 28.9, β = 96.2	*P*2_1_	1.8	3l45	2.6	Sukumar *et al.* (2010 ▸)
CA–acetazolamide	*a* = 42.8, *b* = 41.8, *c* = 72.9, β = 104.3	*P*2_1_	2.0	4g0c	2.0	Fisher *et al.* (2012 ▸)
CA, pH 7.8	*a* = 42.8, *b* = 41.7, *c* = 72.9, β = 104.6	*P*2_1_	2.0	3tmj	1.7	Fisher *et al.* (2011 ▸)
CA, pH 9.0	*a* = 42.6, *b* = 41.6, *c* = 72.8, β = 104.6	*P*2_1_	2.0	3kkx	1.2	Fisher *et al.* (2010 ▸)
CA apo, low pH	*a* = 42.7, *b* = 41.7, *c* = 72.9, β = 104.7	*P*2_1_	2.0	4y0j	2.0	Michalczyk *et al.* (2015 ▸)
Crambin	*a* = 22.8, *b* = 18.8, *c* = 41.0, β = 90.9	*P*2_1_	1.1	4fc1	4.0	Chen *et al.* (2012 ▸)
DHFR–methotrexate	*a* = *b* = 93.1, *c* = 73.9	*P*6_1_	2.17	2inq	0.3	Bennett *et al.* (2006 ▸)
DFPase	*a* = 43.4, *b* = 83.3, *c* = 87.5	*P*2_1_2_1_2_1_	2.2	3byc	0.43	Blum *et al.* (2009 ▸)
Endothiapepsin	*a* = 43.0, *b* = 75.7, *c* = 43.0, β = 97.0	*P*2_1_	2.0	2vs2		Coates *et al.* (2008 ▸)
Z-DNA	*a* = 17.9, *b* = 30.6, *c* = 44.6	*P*2_1_2_1_2_1_	1.4	3qba	0.7	Fenn *et al.* (2011 ▸)
Deoxyhemoglobin	*a* = 63.8, *b* = 84.4, *c* = 54.3, β = 99.3	*P*2_1_	2.0	3kmf	20	Kovalevsky *et al.* (2010 ▸)
Equine cyanomet hemoglobin	*a* = 108.9, *b* = 63.2, *c* = 54.7, β = 110.75	*C*2	2.0	5c6e	10	Dajnowicz *et al.* (2016 ▸)
Photoactive yellow protein	*a* = *b* = 66.8, *c* = 40.9	*P*6_3_	2.5	2qws	0.79	Fisher *et al.* (2007 ▸)
XI apoenzyme	*a* = 93.9, *b* = 99.7, *c* = 102.9	*I*222	1.8	2gve	8	Katz *et al.* (2006 ▸)
XI–Ni^2+^–linear sugar	*a* = 94.0, *b* = 99.7, *c* = 102.9	*I*222	1.8	3kco	50	Kovalevsky *et al.* (2010 ▸)
XI–xylulose	*a* = 94.6, *b* = 100.0, *c* = 104.0	*I*222	2.2	3cwh	4	Kovalevsky *et al.* (2008 ▸)
XI–Cd^2+^–cyclic β-arabinose	*a* = 93.9, *b* = 99.7, *c* = 103.0	*I*222	2.0	4qdp		Langan *et al.* (2014 ▸)
XI apo, pH 5.9	*a* = 93.9, *b* = 99.5, *c* = 103.0	*I*222	2.0	3qza	9	Kovalevsky *et al.* (2011 ▸)
Xylanase, pD 8.9	*a* = 49.6, *b* = 60.0, *c* = 70.5	*P*2_1_2_1_2_1_	1.7	4s2h	7	Wan *et al.* (2015 ▸)
Xylanase, WT–MES	*a* = 49.2, *b* = 60.2, *c* = 70.4	*P*2_1_2_1_2_1_	2.0	4s2d		Wan *et al.* (2015 ▸)
Xylanase, N44D mutant	*a* = 49.2, *b* = 60.3, *c* = 70.5	*P*2_1_2_1_2_1_	2.0	4xpv		Wan *et al.* (2015 ▸)

**Table 3:** Detector specifications Source: Brookhaven National Laboratory.

Active area	1.5 m × 20 cm (8 segments)
Angular coverage	120° (curved) in *x*, 15° in *y*
Radius of curvature at anode (cm)	72.8
Position-encoding method	Resistive charge division
Position-decoding method	ADC, FPGA and DSP center-of-gravity calculation
Event processing time	∼4 ms per segment
Event timing resolution (ms)	∼1
Rate capability (global) (s^−1^)	≥10^6^
Gas mixture	7 atm ^3^He + 2.5 atm propane
Gas depth (cm)	1.5
Nominal gas gain	50
Readout channels	15 in *x*, 16 in *y* per segment
Readout node spacing (mm)	12.7
Wire pitch (mm)	1.6
Position resolution	1.5 mm FWHM for thermal neutrons
Image size (pixels)	480 × 512 (245760) per segment, 1966080 total
Detection efficiency	∼50% at 1 Å, >90% at 4 Å
Weight (kg)	∼250

**Table 4:** Macromolecular neutron crystallography instruments

Instrument	Location and source
LADI-III	ILL, reactor
D19	ILL, reactor
MaNDi	SNS, spallation
IMAGINE	HFIR, reactor
BioDiff	FRM-II, reactor†
iBIX	J-PARC, spallation
BIX-3	JRR-3, reactor
BIX-4	JRR-3, reactor
PCS	Lujan Center, spallation

† FRM-II uses a monochromatic beam and most often an IP, with a CCD option.
